# Plural media ethics? Reformist Islam in India and the limits of global media ethics

**DOI:** 10.1007/s10624-021-09626-5

**Published:** 2021-06-19

**Authors:** Max Kramer

**Affiliations:** Institut für Ethnologie, Ludwig-Maximilians-Universität München, Oettingenstraße 67, 80538 München, Bavaria Germany

## Abstract

The transatlantic field of global media ethics is premised on a search for the conceptual foundations of plurality. This article is a critique of this very endeavor. I offer this critique through works authored by moral anthropologists of Islam and through a close reading of the Urdu text *Cyberistan: Muslim Naujavan Aur Social Media* (Cyberistan: Muslim Youth and Social Media) authored by Sadatullah Husaini, the current president of the Indian reformist Islamic organization Jamaat-e-Islami Hind. My article is a post-foundational critique of the implicit foundationalism through which “Islam” and “plurality” are related to each other within inquiries into the ethics of digital communication. I take on digital communication because of its increasingly global and synchronic nature that rendered questions concerning plurality in media ethics particularly urgent. I argue that even though it is important to ask what difference means conceptually for a global media ethics today, it can only make space for radical plurality via the negative, by way of its contradictions and structural constraints. If a global media ethics is supposed to be based on openness and plurality, it can be so only by limiting and weakening its own ontological claims – beyond positive metaphysical groundings, cultures, civilizations, Islam, etc. In other words, it requires a reflexivity to its own position as an academic discipline that produces knowledge under certain historical conditions and an understanding of its own political practice.

## Introduction

Is there a global digital media ethics? Surely not as an established discipline or a practice that would connect citizen journalists who are, say, from India with those in France. Surely not as an approach or a shared authoritative reference that would trouble the consciousness of online trolls from Bangladesh to Norway. I am certain that many people who lead their lives increasingly online and observe the new scales of global connectedness may have wondered how to act regarding the diversity of values, norms, and virtues required to blaze a trail through the wilderness of digital communication. However, there also exists a field of inquiry – tethered to the disciplines of communication and philosophy – called “global media ethics” which has recently engaged with questions of the digital realm. The key arguments this field proposes concern the global world in continuation with discussions in journalism ethics. The digital condition poses new questions concerning the convergence of what were formerly more separate media and the participation of all kinds of (non-professional) agents in the production of news (Ess 2014; Ward 2015; Christians 2019). It also makes us question the terms of our everyday participation within increasingly digital mediascapes. Many scholars think that the digital realm further urges us to come to terms with the aforementioned global diversity of norms, values, and expectations. To cite Charles Ess, who published a popular textbook on the issue of “Digital Ethics”:

[…] digital media as global media […] force us to confront culturally variable views – regarding not simply basic ethical norms and practices but, more fundamentally, how ethics is to be done. In particular, we will see that non-Western views – represented in this volume by Confucian, Buddhist, and African perspectives – challenge traditional Western notions of the primary importance of the individual, and thereby Western understandings of ethical responsibility as primarily individual responsibility. (Ess 2014, 21)

Indeed, these are important questions and they merit investigation. Notice that, while “the West” is represented by liberalism and “culture” serves as the crucial dimension of ethical plurality, Islam is not among the approaches mentioned as a potential challenge to Western individualism. In fact, some transatlantic^1^ authors in this field position Islamic media ethics either explicitly or implicitly outside the scope of a conversation on the *ontological foundations* of the topic – by aligning Islamic values and pious dispositions to the available concepts of the field, by not engaging with them, or by claiming they are irrelevant to the discussion. For example, Clifford Christians, a well-known scholar in the field, claims that Islamic media ethics draw on absolutist positions (Christians 2019) by being based on the metaphysical principle of *tauhid* (the oneness of God).

At this point, it is crucial to state that this article is *not* a contribution to Islamic media ethics or to global media ethics. I do not try to define or defend either “Islam” or the “Western” ethical traditions. In fact, both of these terms are often used in problematic and schematic ways within the field. This article is meant as an immanent critique of an academic field whose questions I find important and whose answers concerning plurality I deem unconvincing. The argument is *immanent* because it takes as a starting point the criteria the field sets up for itself as a measure. Therefore, I neither want to add a new positive approach^2^ nor a new perspective (“Islamic perspective”, “negative pluralist perspective”, etc.) to the field but to *point out its contradictions and limit the scope of these positive and normative endeavors*. The critique I want to advance is directed at the *ontological foundations* from which postulated differences of various kinds (primarily of religion and culture) have exclusory effects that run counter to the field’s claims of conceptual openness. By ontology, I mean the basic assumptions people have regarding what kinds of things or processes there are in the world, how they are related, and what structures establish both the things/processes and their relations.

## Global media ethics

Before I come to a larger review on the position of Islam within this body of work, let me briefly introduce some of the key thoughts on ethics and the problem of diversity by three prominent thinkers: Clifford Christians (2013; 2019), Stephen Ward (2013; 2015), and Nick Couldry (2013; 2016). I have chosen these three scholars either because they have come to notice as the editors of volumes on global media ethics or are featured prominently in them.

Stephan Ward stresses the need “to articulate cosmopolitan aims and principles for journalism, such as serving humanity, acting as a bridge of understanding among warring groups, and promoting human rights” (2015, 110–111). For Ward (2013), global media ethics is an open-ended “project” meant to question “parochial” understandings of ethics and reinvent new ones by radically rethinking epistemological and ontological foundations of media ethics within new digital communication environments. Couldry presents a neo-Aristotelian view of ethics concerned with the question of:

how we should act, and second, with how, consensually, to build a framework for thinking about how we should act. Ethics aims at a reasoned and inclusive debate about value, rather than a search for moral absolutes or systematic rational frameworks of moral justification. (Couldry 2016, 109)

For Couldry, the strength of virtue ethics is that no transcendental point of reason or style of reasoning is required. Additionally, he considers it helpful that Aristotelian thought is at some distance from today’s religious ethics, which may enable dialogical positions between them (ibid. 110).^3^ Clifford Christians claims that the primal “sacredness of life” is a “proto-norm” that establishes mutual respect. This proto-norm eventually makes derivable other important values such as human dignity and truth. His conceptual terrain stretches from Christian ethics to Heideggerian existentialism (perhaps not quite taking the latter’s abyss in its radical implication; see Christians 2019). For decades now, Christians has raised important questions that go beyond the liberal textbooks on journalistic ethics – although he often gets caught up in the unproductive binary between what he calls “relativism” and “absolutist” approaches (where, as we have seen, Islam ends up slotted in the latter).^4^

While I do not intend to capture the complexity of the authors’ arguments in their entirety,^5^ I only hope to establish a couple of key features that are central to the way the field approaches ethical questions and its claims to reach towards difference. The points of reference for this endeavor circle around virtue ethics (Ess 2014; Couldry 2016) and humanism (Christians 2013, 2019), as well as non-western approaches that are understood to be combinable with the author’s preferred model as long as they can be regarded as “non-absolutist”. By the latter term, most authors understand this to reference different ways to overcome the utilitarian, autonomist and deontological biases of European enlightenment and liberal thought. To make their points concerning plurality, scholars have been drawing on Ubuntu (Rao and Wasserman 2007; Metz 2015; Christians 2015), Thai Confucianism, and Tao (Gunaratne 2005). Although there are many differences in the three approaches of Couldry, Ward, and Christians,^6^ I want to highlight three common features:

1. The possibility to develop a common conceptual terrain of ethics (to some extend separable from the political^7^) to ground a pluralistic – sometimes consensual – understanding of global media ethics.
2. This positive framework may enable or enhance dialogue between different ethical frameworks, concepts, principles, and practices.
3. A commitment to think beyond liberal norms of journalistic ethics is important to open up questions of being, relationality, and pluralism in times of radical socio-technological change (especially concerning the challenges of digital communication technology).

These three dimensions are, indeed, part of an important response to global transformations within the current times of information/surveillance capitalism (Zuboff 2019) and the globalization of moral panics surrounding the digital (Ess 2014, 3). I am interested in the way these attempts by transatlantic scholars arise in the form of theoretical principles or foundational frameworks that, they claim, have not been derived from *one* normative source of authority (which would be moral absolutism). Instead, they suggest that if we want to figure out a global media ethics *together*, we *should* rationally adopt the approach suggested. In other words: we *should* accept the respective scholars’ understandings of ethics and pluralism (or at least the plurality of approaches to virtues and values) – hence, we *should* accept that one can build ethics as a separate sphere of intellectual activity on its own terms as far as it concerns cultural and religious difference. To quote Christians on this: “Ethics is a world of ideas and therefore foundations and intellectual substance are inescapably important” (Christians 2015, 61). With this approach, we are, however, failing to appreciate the way *our relation towards God, capitalism, our bodies, political struggle, and so on, may be crucially co-constitutive of ethics as well as socially situated*.^8^

Stephan Ward (2013) distinguishes four types of studies: empirical, empirical-normative, applied, and philosophical. It is important to mention the fourth type here because it is about ontology (about foundations):

The fourth type of inquiry works at the meta-ethical (or philosophical) level. Inquirers examine the theoretical foundation of global media ethics. Foundational topics include the goals and basic principles of global media ethics, the existence of media universals, and the challenge of ethical relativism. (Ward 2013, 4)

The problem is this: The very division Stephan Ward makes between the four types of global media ethics renders it difficult to find out how concepts are related to and implicated in social life (assuming that there would otherwise be no need for them in the first place). This is why, instead of squarely placing this article within Ward’s fourth level of analysis, I will try to create a field of conceptual force that is *inside global media ethics enough to confront it with its outside*. In other words: I will point out gaps and contradictions within a set of frameworks that are *connected by the impossibility to build their plurality on the very foundations suggested (including the idea of foundations)*.

Let me now come to my two main arguments. First: Different elaboration of ethical principles, values, duties, virtues, and recommendable behaviors need to be explored via the institutions and traditions which produce them and within which they may have some grip on social reality. This contrasts with some of the work in the field that elevates ethical principles to rather abstract speculations on “Islamic contexts” and “civilizations”. In the second part of this paper, I will show one way to avoid such a handling of Islamic media ethics by referring to the writings of Sadatullah Husaini, the current president of the Indian reformist Islamic organization, Jamaat-e-Islami Hind (JIH). I argue that the aim of discussing “ethical principles” is already compromised by a post-enlightenment premise. Rather, I read global media ethics against anthropological studies that analyze the relation between morality, religion, and mediation (the human practices that involve communicative technologies; e.g., Asad 2003; Mahmood 2005; Hirschkind 2006; Lambek 2012). Through their engagement with mediation practices by which morality gets embodied and negotiated in everyday life, they challenge a number of assumptions that are common within media ethics literature – including the very stability of the terms *religion*, *ethics*, and *morality*. The anthropologist Michael Lambek (2012), for example, does not differentiate between morality and ethics. This old Aristotelian fault line connects much of the work within global media ethics with post-enlightenment philosophy that regards *ethics* as a “systematic study of people’s reasoning when they are said to act morally” (Mahmood 2012, 224). Within this rather dominant framework of ethical thought, morality points to “collective rules, norms, and codes” (Mahmood 2012, 223). Hence, much of global media ethics already sees through a thick lens that distances the body from ethical rationality, the public sphere from religious intensities, and principles from pious dispositions. But even if more cohesive attempts at grounding are made, the assumption still rests on the possibility to come up with a *common ethical framework* phrased by Hamada in the following way: “Without such universal ethical grounds, the alternative is the inevitable chaos. The challenge involves establishing a universal ethical model that both protects and limits ethical pluralism […] (Hamada 2016, 2). My contestation is precisely that this “universal ethical grounds vs inevitable chaos” is a false alternative. To save plurality in ethics, one has to engage with radical difference. This means one may need to limit exactly this consensual foundationalism itself and relate it to its own moments of institutionalization (the political), its own social grounds, and its history.

Hence, my second argument is as follows: Even though it is worthwhile to ask the question of difference for a global media ethics of digital communication, it can only be via the negative, by way of its contradictions and structural constraints. Here, it forms part of an alterity that needs to be acknowledged on an ontological level, where it cannot be measured against transatlantic frameworks of ethics. On this level, cultural and religious differences are at once enmeshed in the political; they cannot be resolved through consensus or, indeed, any positive ethics. However, global media ethics has an important place as one productive conceptualization of the good that elaborates upon some of the real challenges of coming to terms with the global transformations of information capitalism. From its elaborations, we learn a great deal about possible ways of questioning our online behavior; however local these questions may turn out to be.

### I

## Media ethics and Islam

I will begin by reviewing some contributions within the larger field of global media ethics and communications that did engage with Islam in a more sustained fashion. Since these discussions of journalistic and mass media ethics on a global scale prefigure the thinking on digital ethics, it is worthwhile to introduce them here, however, briefly. Mohamed (2010), in his contribution to *Media Ethics Beyond Borders* (ed. by Stephan Ward and Herman Wassermann), mirrors reformist Islamic discourse (Hartung 2013) on Islam being “a total life system” (Mohamed 2010, 144), an “integrated relationship between religion and politics” guided by principles directly derivable from the normative sources of the Quran and the Sunnah.^9^ His elaborations seem to be premised on “Islam” being somewhat *too late*, asking: “To what degree is Islam compatible with global human rights and global journalism ethics in general?” (Mohamed 2010, 145). Even though Berenger and Taha (2013) concentrate on the repression of “free speech” in different Arab states, when they turn to “Islamic contexts” (ibid. 93), we have a similar approach to Mohamed (for they take reformists such as Fazlur Rahman as representatives of “Islam” as such). The authors draw on separate quotes from the Quran that were meant to direct Muslims towards diverse values such as “truth”, “freedom of speech” (with its limitations regarding ridicule), and “dignity”. Similarly, Bassam Tibi contrasts an ideal affair of global communication against what he calls a “realist” understanding of cultural fragmentation and “identity politics of we versus them” (Tibi 2011, 57). Tibi measures whatever he finds lacking in “Islamic civilization” – or even “Islam” – against the question: “Could the discourse of cultural modernity be shared as a means for global communication?” (ibid., 57). It must be clear that no fine-grained understanding of moral and ethical thought may ever escape the overbearing dichotomies that drive such premises.

The problem with these texts is that they assume an “Islamic context” (Mowlana 2007, 24; Mohamed 2010, 145; Berenger and Taha 2013), or a “civilization” (Tibi 2011) based on infinite regress (contexts of contexts of contexts of contexts of “Islam” that pull it together whenever it fits “principles”, and “worldviews” and “ways of life”, etc.) to be stopped only by juxtaposition with a moral-political boundary. The latter is mostly liberal (and/or cosmopolitan) reasoning, situated as either its premise or its designated other. Another interpretation that founds media ethics via “Islam” and responds to some of the conundrums of the global rise of citizen journalism and convergent digital media cultures is put forward by Hamada: “The model is based on four guiding principles: respecting pluralism and cultural diversity, freedom of expression, justice, and moderation. What distinguishes the Islamic ethical model is its human universal ethical values that should be given priority over political partisanship, national and personal interests, or technological determinism” (Hamada 2018, 50). Hamada’s model is meant to challenge “western bias” and can be read in continuation to the foundational endeavors of global media ethics.

Here is not the place to elaborate on all the possible ways to understand Islam in relation to media ethics.^10^ It would have been helpful if some of the above-mentioned authors (e.g., Mowlana 2007, Mohamed 2010) clarified that they are referring to different forms of modern reformist Islam. In this regard, scholars have pointed out that ethics are not necessarily driven by abstract principle but rather directed to the formation of virtuous selves that may transform the political community through moral debate, dispositions, and action (Hirschkind 2006, Ahmad 2017).^11^

Outside these overtly normative approaches, there exists a body of empirically driven studies that are interested in editorial practices and moral values among Muslim journalists or in Muslim countries (Hafez 2003; Pintak 2014; Hamdy 2016; Issawi 2016; Steele 2018). They compare practices and values within different nation-state polities and with western journalistic standards. Lawrence Pintak, for example, organizes his comparative and survey-based study of Muslim journalists around core values (truth, justice, and independence) which he relates to diverse textual traditions (with an eclectic range stretching from the modern reformist Abul Ala Maududi to the medieval jurist Ibn Qayyim, on to direct quotations of the Quran). Janet Steele’s recent book (2018) on Islam and journalism in Indonesia and Malaysia merits special mention here for its degree of ethnographic detail and empirical insight. She points out that – even though the normative discourse is voiced through Islamic perspectives – the actual core values between western journalism and her field of informants largely overlap. She speaks of “para-ideologies” (ibid. 17) to point out that “western media” often makes a divide along liberal lines, between enlightenment and fundamentalism, while Islamic approaches center on justice, it being legitimated through references to Sunnah and the Quran. While criticizing western biases, she attempts to balance things out by retreating from some of the instabilities and gaps between theory and practice (Hafez 2019). This point was made in a review of Steele’s book by Kai Hafez:

It is true that the reality of Western media coverage is a long way from objectivity, especially when picturing Islam mainly as negative or blending out Muslim voices. However, it is also true that this has nothing to do with the formal ethics of secularism, but is a flagrant violation of the rules of objectivity and balance. One should criticize Western journalism for its often-ill-reflected Orientalist, culturalism, or even racist practical biases, not for its secular-egalitarian theory. (Hafez 2019)

Hafez is right in arguing that there are gaps between theory and practice. He, however, may not appreciate the problem that the secular-liberal discourse is also deeply steeped in morality (of a transcendental kind of reasoning in the widely received works of Rawls and Habermas). Liberal secularism (perhaps less than egalitarianism) has been shown to confront difficulties in the attempt to accommodate alterity since it construes its religious other in need of regulation as an afterthought to its own logic (Asad 2003; Mahmood 2005; Hirschkind 2006; Scott 2007). It also runs into trouble if its other does not measure up to liberal ideas of consensus or tolerance (see, e.g., Brown 2008) or if it cannot be captured within legalist and procedural categories (Mouffe 2000).

In fact, what marks most of the above examples from transatlantic academia is the fact that they *do not* differ much in their literalist approach towards “Islamic principles” and “values” from a body of Islamic missionary sciences (*dawa*)^12^ in English – sometimes similarly decontextualized from their social-material force and traditions (or the force is precisely an academic reproduction, in which case they would instead be divorced from Islamic traditions). Before I turn to this body of works, I have to acknowledge that there would likely emerge a rather vast corpus of literature on “*dawa* and social network sites” in Southeast and South Asian languages, Persian, Arabic, and Turkish. To this corpus (besides the few texts in Urdu that I will examine below), however, I simply do not have access and nor was I able to find any study that would refer to such works in any language which I know.

In the year 2008, Al-A’ali claimed that the relationship between the ethics of information technology and Islam has received little to no attention – a position which was repeated by Abdul Kadar in 2013. To me, several interview partners of the JIH confirmed this claim. Of the available English language literature, it is safe to say that Islamic media ethics have been developed in the field of journalism. Only recently have a number of English articles appeared to deal with social network sites (SNS) as particular sites for ethical reflection and guidance (Norwawi et al. 2014; Fauzan 2013; Rusli 2013). From these articles and others in the field of computer ethics (Al-A’ali 2008), I have gotten the impression that there is a fluid boundary between social science scholarship and moral advice, both converging on the articulation of “Islamic perspectives” generated by referring to the authoritative sources of the Quran and the Sunna (sayings and behavior of the prophet). The texts are also sprinkled with some literature that helps to position them within certain fields of academia (e.g., computer ethics, journalistic and media ethics, missionary science). The authors draw on transatlantic scholarship to derive additional material for pointed criticisms on the negative effects of social networks.

Therefore, the problem in a larger comparative endeavor would be to unearth and cross-examine the moral discourses of Islam relating to media in their different socio-cultural and historical contexts. In other words, one would need to study how they were articulated through specific discursive traditions (Asad 1986), and within institutions that are often tethered to modern nation-state polities and to political economy (see, e.g., Islam 2015; Iqtidar 2011; Yavuz 2003). Thus, neither religion nor ethics can be pre-stabilized as a specific realm of symbolic and moral world-making. The categories rather require discussions to be historicized and located within specific traditions that develop particular styles of reasoning in the way they “instruct practitioners regarding the correct form and purpose of a given practice” (Asad 1986, 14). More radically, Talal Asad (2003) has shown that religion as such is a modern concept always pitted against the secular; concerns within discursive traditions, however, often crisscross these modern fault lines.

### Post-colonial media ethics?^13^

I must discuss the work of Shakuntala Rao and Herman Wasserman (2007) briefly because it has raised some similar concerns to my own. The authors profess that theories have so far been derived from western conceptual sources and have not taken in “ethical principles” from post-colonial perspectives. They importantly notice the absence of the political (ibid. 30) and want to highlight how cultures are “enmeshed in power relations” (ibid.) that need, partially, to be seen in continuity with the global spread of capitalist modes of production (ibid. 34). Drawing on the language of hybridity and cross-cultural comparison, they aim to unearth the conditions of post-coloniality in which “principles are to be applied” (ibid. 31) by questioning the relationship between western (often liberal) assumptions about social life and their unreflected and non-reciprocal export to non-western places. Part of the claim is that there is culture-based difference related to ethical principles that should be given the status of “theory” (ibid. 36):

Thus, concepts such as *dharma* or *ahimsa* from Indian philosophy, for instance, are used as evidence to construct and validate larger notions of social responsibility theory […]. *Dharma* and *ahimsa* do not attain the privileged position of theory but are relegated to evidentiary materials. (ibid.)

This implies a questioning of Euro-American theory by the margins in a dialectical movement that provincializes the center (ibid 37). It is directed to change and disrupt “patterns of power not merely with the incorporation of different points of view in order to reach consensus” (ibid). Shakuntala Rao frames the overall project in the following way:

I focus on the ways postcolonial critique gives us possibilities to expand on what we define as ethical theories and conceptual frameworks. Ultimately, it is an effort that Appiah [2006, p. 94] calls the acknowledgement and “respect for historically, socially, and politically authentic identities of others” to which I add the recognition and respect for theoretical identity of others. […] One task of the postcolonial theoretical project has been to recover the systems of knowledge which have been a priori considered outside of modernity but are deeply implicated in modernization and the modern subject. (Rao 2010, 94-95)

While I agree with Rao’s second argument, the former (for which she quotes Appiah) is premised on an understanding that seems to assume cultural differences as a good in itself, instead of as a response to a problem faced by social agents (answered by identity formation, political demands, ethical openness, etc.).^14^ This position conflicts with attempts to consider the political together with the ethical because certain entities like Indian philosophy are pre-stabilized and then pitted against western theory. The problem here is that *Gandhian* terms are metonymically sliding into “Indian” philosophy as a supposedly marginalized voice. Here, differences are overdetermined by the hegemony of a cultural container, India (or in the case of Ubuntu, “African thought”; see Rao and Wasserman 2007, 40).^15^ This can be shown just by asking ourselves how a thinker like Sadatullah Husaini of a reformist Islamic organization could qualify as an Indian philosopher. Certainly, he is an Indian citizen working within an Islamic organization that articulates its politics largely towards the Indian nation-state, but does that render a concept or technical terms he uses as “Indian”?^16^

Thus, this post-colonial approach^17^ prestabilizes the categories it wants to critique. In other words: It arrives on the scene both *too early* (it already has “local cultures”) and *too late* (it re-acts to the “West” or “Euro-America”). Even if Foucault – who is quoted as a theoretical reference in Rao’s text – would have been elaborated upon regarding historical singularity (Veyne 2009), the author could have steered clear from this dialectic of “cultures”.^18^ Instead, the kind of questions I want to raise are: Where do “theories” – as in their location in transatlantic academia – hit the boundaries of other moral traditions by becoming stabilized (and commodified) in categories such as “global media ethics” or “post-colonial theory”? Even though the relationship between different conceptual traditions is important, it is not a goal in itself to have some culturally localizable knowledge but rather a question of how to respond to an urgency that makes us reflect and comport ourselves ethically/morally. Why is it that we assume that this is a job which can be dealt with in an academic discipline – intellectually advanceable on its own turf?

### Limiting ethics

To sum up: My understanding is not premised on either some foundation or some reaction to “western theory”. If ethics is to encounter the open, we should give it some space. This includes the acknowledgement of the contingent boundary between morality and ethics which is itself a political question. Contingency refers to historical openness, the simple fact that it could be otherwise and could have been otherwise while acknowledging that things have nevertheless become the way they are.^19^ Post-foundationalism is the paradox of the necessity of thinking in ontological terms (e.g., founded on God, reason, culture, or history) while rejecting their claim to universality^20^ (Marchart 2013). Thus, contingency involves political struggle since differences are not solvable only by reason or consensus. Now, the same should be true for ethics, as it is desired by global media ethicists: Our way to open to the newness brought on by an encounter with something which is truly different. Jürgen Schaflechners’ deduction reveals the contradiction of *grounding* a post-foundational approach while clarifying the conceptual stakes for the question of plurality in media ethics:

I start with its first part, the impossibility of an undisputed foundation that grounds society [read here a “pluralism”; MK.]. If this statement holds true, we encounter a contradiction – namely, the claim that there can be no single foundation becomes itself a foundational claim. In other words, the absence of any last foundation needs to pertain to *all* foundations, which produces a double bind. If we are to accept the plurality of foundations (or ontologies), then this plurality is valid for all and, hence, a foundational principle. If we do not accept the plurality of foundations, however, then some universal grounding fact needs to be accepted […]. In other words, the moment we aim to criticize foundationalism, we are already in the process of creating another foundation. This reveals how the assumption of an absence of any last foundation *necessarily* leads to the creation of another foundation. (Schaflechner 2018, 25)

If a global media ethics is supposed to be based on openness and plurality, it can be so only by limiting and weakening its ontological claims – beyond positive metaphysical groundings, cultures, civilizations, etc. In other words, it requires a reflexivity to its own position as an academic discipline that produces knowledge under certain historical conditions. This positioning may enable media ethics to open up to other discursive traditions that have an intellectual field of force of their own. By this, I mean that they are not necessarily developed as answers to particular problems faced by post-colonial theorists or global media ethicists (e.g., searching for “principles”, challenging “universalism” and “absolutism”, or developing “theory”). When foundational conflicts are taken seriously, their recognition may help to decenter ethics and philosophy along with it. Thus, a weakening of ontological claims could, on the one hand, strengthen dialogue between moral traditions for which the moral stakes could be reconstructed closer to their own grounds. On the other hand, moral transformations may be imagined and studied through political contestations that go beyond discursive traditions as they exist so far. Politics can here be understood as limiting and institutionalizing moral orders (Glynos and Howarth 2007). My approach to immanent critique broadly resonates with post-structuralist approaches in the sense that political contestations do not have to come completely from within different moral traditions (like communitarians such as Michael Walzer would have it) but always transcend those traditions in relation to a problem faced (e.g., problems that erupt between political institutions, economics, ecosystems, and technology).

In the following section, I will demonstrate my argument by discussing the booklet “Cyberistan” on moral guidance concerning the Internet, written by Sadatullah Husaini (2018), the current president of the Jamaat-e-Islami, Hind. I have chosen this text for two reasons. First, “Cyberistan” must be considered a rather influential text among cadres of one of South Asia’s biggest reformist Islamic organization and therefore may exert some interest in its own right. Second, as I have shown above, even though some conceptions of reformist Islam have found their way into the field of global media ethics, other discursive traditions of reformist Islam have developed a rather different moral framework while engaging with similar problems. Husaini’s approach remains at some distance from the foundational concerns of the transatlantic field of global media ethics. This distance is worthwhile to measure. As a disclaimer, I need to add that my knowledge about non-South Asian reformist Islam is rather limited. Therefore, I am not the scholar who could assess Arabic and Persian sources of reformist Islam (such as Mohammad Abduh or Muhammad Rashid Rida) and would be able to reconstruct longer genealogies and global connections between reformist traditions as they pertain to the development from journalism ethics to the ethics of digital communication. However, I think my argument still stands as it is primarily concerned with the *possibilities of the concept of pluralism* within the questions and problems raised by the field of global media ethics.

### II

## Dawat and the nation

The Jamaat-e-Islami is an organization that engages in *dawat*, an Urdu term derived from the Arabic *dawa* that literally means “invitation” in the sense of the proselytizing, preaching, and propagation of Islam. *Dawat* practices are meant to bring about moral and political transformations in the sense that they are directed both at the development of pious subjects as well as on the transformation of the polity into a more Islamic one. They have been shown to build a particular public sphere in which discursive participation within the *umma* (the Islamic community) is “also grounded in political technologies of modern national citizenship” (Hirschkind 2006, 37). Hence, *dawat* has become “a critical site for the expression of those demands engendered by political modernization” (ibid. 38). As opposed to its sister organizations in Pakistan and Bangladesh (Iqtidar 2011; Islam 2015), the Jamaat e Islami in India (Ahmad 2009) does not partake directly in electoral politics. It rather acts in the field of education and as a political pressure group. For this purpose, the organization runs several media teams in different Indian states as well as at the headquarters in New Delhi. The context of being a minority organization has driven the JIH to accept the framework of state secularism as one in which claims and demands upon Muslim participation can be put forward (Ahmad 2009). While the Jamaat is overtly critical of the current Hindu-nationalist government, leading personas within the party are nevertheless careful not to challenge secular nationalism. They rather appropriate the overall legalistic discourse of Muslim minority politics in India (Ahmed 2019). Although the party officially engages in politics online, it does so through a highly guarded discourse that arguably pertains both to its vulnerability as a minority institution engaged in proselytization^21^ and to its overall moral outlook on participation in public discourse.

### Cyberistan^22^

Sadatullah Husaini was elected as the president (*amir*) of JIH in 2018. Formerly, he was the national president of the Jamaat’s youth wing, the Students Islamic Organization of India (SIO). He upheld several functions in the party, including being a board member of the Majlis-e-Shura (the consultative assembly) and heading the JIH’s Study and Research Department. Besides his functions within the Jamaat, Husaini is the director of several social organizations and a board member of several educational institutions. His academic background is in electronics and telecommunication engineering, and he has authored several books in Urdu which deal with questions of globalization, imperialism, post-modernism, and technology.

The booklet “Cyberistan” (2018) consists of two chapters that were written years apart.^23^ The first and earlier chapter follows an academic format, including references and endnotes, while the second is a more essayist piece of moral advice literature. Both chapters are concerned with social and technological change as related to moral comportment and virtuous dispositions. The text’s Urdu addresses primarily party cadres as well as the large network of supporters of the Jamaat, many of whom can read and write Urdu proficiently (while Hindi is more common among younger Muslims in India today due to the lack of state funding and career opportunities regarding Urdu). The claims the book advances are, however, meant to be universal to the *umma* (hence, potentially, to human kind since everybody could become a Muslim one day).

Husaini states in his preface to the book that fast social and technological change can lead to a condition in which many normal people may be led astray. Thus, the book should be a “guidance in matters of required (*matluba*) behavior”. He stresses that although there are plenty of Islamic websites and online activities authored by Muslims, very few serious attempts to create an “Islamic perspective” (*islami nuktanazar*) on cyberspace have so far emerged (Husaini 2018, 8). Husaini mentions some of the positive sides of the Internet when it comes to Islam: the transnational nature of the net, its cheap availability, the chance to get around gatekeepers while speaking truth to power, and, finally, the possibility of bringing people together from around the world in order to spread Islam. However, in most of the two chapters, he instead concentrates on the challenges and vices that emerge with digital technology and social networking sites.

He begins the first chapter with a division between reality and image: People often search for refuge from the bitterness of life in “valleys of beautiful dreams” (ibid. 7). The image realm of “Cyberistan” is filled with artificial desires in which “the distances between the real reality and the artificial reality disappear” (ibid.). This happens when man is searching to create his subjectivity on the autonomous basis of individual desires. While the Internet may be a useful technology, “Cyberistan” is not (ibid. 9). When imperialist capitalism approached its accumulative frontier, “Cyberistan” became a new ground for the creation of wealth (ibid. 8) while reproducing some of the individualist fallacies of the West.

The second essay deals with a relationship between organized social struggle (*samaji jihadkari*) and the use of social media. Husaini stresses that there is a difference between slacktivism and the “struggle on the ground”. The offline interaction between two human beings is phenomenologically richer, involving individuals’ “aura” and a propensity to experience emotional warmth and empathy. For this reason, social media should serve the offline life instead of taking its place (ibid. 26). Most importantly, there needs to be a clear purpose (*maqsad*) for any online activity since Muslims need to portray exemplary behavior (ibid. 27). Husaini mentions, again and again, that people should not waste time by engaging in unserious activities.

Finally, his guidance includes suggestions such as “draft your monthly and yearly goals for social media use and limit your activities to the achievement of those” (ibid 29). There should be no unnecessary details on your profile, so that people cannot see your family pictures in public. In the “real life”, there are different rooms and private spheres where different content can be communicated and different forms of behavior are allowed and domain-specific – for example, among friends, in the bedroom, in an organizational protest march, or in a public address. These domain specifications should be maintained on social media as well (ibid. 30).

Another point he stresses is that one should do some research before forwarding a post. He urges readers to remember the *hadith* [sayings and behaviors of the prophet] that “for man to become a liar it is enough to relate a story to others without having done any inquiry” (ibid. 30). Since social media is a powerful tool to spread information, it can become dangerous if used for misinformation. Husaini urges readers to remember that “we have to give an answer in God’s presence for every uttered word”.

The last point I would like to mention is that *dawat* activities should be conducted with wisdom (*hikmat*) and through thoughtful debate. One should not abuse the deities of non-Muslims. The missionary’s work is not to force the message on people but to pass it on peacefully. If, after all arguments have been presented, the other is still not convinced, one should rest the conversation with emotions controlled (ibid. 31).

### Moral advice literature and modern self-making

“Cyberistan” is moral advice literature directed at improving the practice of Islamic proselytization within the institutional setting of a reformist Islamic party in India. Although classical *adab* literature (Robinson 2013) was directed at diverse readers including princes, musicians, housewives, and Sufi saints, the specific form of virtuous self-making articulated in Husaini’s text is informed by the notion of an emerging middle-class’ individual’s “self-discipline” with his duties and piety outside of the traditional aristocracy. After their association with Muslim rule came to an end in the mid-nineteenth century, Islamic scholars became increasingly dependent on Muslim society for support. Hence, the question of “how to be a Muslim” came up, making individuals themselves responsible for their soul and the well-being of their community. In this process, values of self-perfection, restraint, fulfilling one’s duties, and being reliable and orderly became increasingly important among emerging Muslim middle classes (Pernau 2013, 242–276). They were also professed by Abul Ala Maududi, founder of the Jamaat-e-Islami (the party of Islam), who needs to be positioned in an ambivalent relationship with the Islamic traditions of religious learning like the madrasas, Sufi brotherhoods, and schools of jurisprudence (Hartung 2013). His approach was to found the polity on God while directing his research and intellectual energy towards what he considered the most fundamental sources of moral order in Islam, the Quran, and the tradition of the prophet (*hadith*). He was interpreting them in the light of modernity with its new technology and political ideologies. The Jamaat became a cadre-based party, a Lenin-style avantgarde that worked as a social activist organization in relation to both the Muslim community and the non-Muslim majority in India. This activism is matched by the thrust of moral advice literature to overcome the dualities of knowing and acting (Ahmad 2017), characteristic of *adab* as a combination of codes of behavior and methods of personal formation.

Husaini’s advice on virtues, vices (*fitne*), and appropriate behavior (*matluba ravaye*) draws in the reformist fashion of Maududi on western sources: internet skepticism has been widely received by him. Perhaps his most central reference is the work of Ziauddin Sardar (1996), particularly his edited volume “Cyberfutures” from 1996. Supposedly, the title of Husaini’s book is directly taken from Sardar’s term “Cyberia” (a word that gives the sensation of an immediate chill of cold air). Sardar argues that there are parallels between the colonization of cyberspace and the subjugation of the colonial world – saturated by colonial metaphors, a quest for new markets and frontiers. The cyberspace figures for Sardar as the West’s dark Other, taking on a reality of its own and projecting back its “untamed, […] psychotic inner reality” (Sardar 1996, 15). Certainly, Sardar’s text converges largely with the Jamaat’s overall positioning towards a cultural and moral critique of western imperialism and capitalism. Sardar also features the differentiation of reality proper and virtual reality linked to a critique of postmodernism. For Sardar, the latter term refers to the idea that everything can be constructed when life becomes an image without an original. Today, this differentiation seems rather dated, with scholars highlighting the deeply mediated nature of everyday life (Couldry and Hepp 2017). Many global media ethics scholars conceive of an ethics as part of our life-worlds that have become unescapably saturated with the images of media technologies and are therefore in need of being linked to non-instrumentalist approaches such as virtue ethics (Couldry 2016), existentialism, and relational approaches (Ess 2014; Christians 2019).

Husaini – even in his second piece from 2018 – seems much more positive than Sardar and the Internet skepticists (e.g.,Turkle 2011), articulating an instrumentalist understanding of social media – moving away from those who try to think of online culture primarily in terms of environments or, like Sardar (1996, 16), as a structurally tainted imperialist terrain. If one follows Husaini’s guidance, a better online culture is possible without the condition of radical structural or material reform. This approach is not uncommon among scholars (*ulema* and reformist) talking or writing about social media in Urdu, as can be seen from a recent interview with Mufti Omar Abedden Qasmi Madni on the YouTube channel *al Marjan*,^24^ or an essay posted on the Facebook page of the JIH by the Maharstrian chapter’s senior member Salim Khan, this work commenting on the use of WhatsApp as primarily a “means of transport and transmission”.

At this point, I want to advance a sociological argument: The piety and practice of self-restraint articulated by Sadatullah Husaini could be read in the framework of the contested nature of *dawat* practices in a minoritarian setting, while political parties without a *dawat* agenda can mobilize in altogether different ways, scales, and digital intensity (see same author 2021, forthcoming). It is impossible to ignore the nation-state framework within which an Islamic discursive tradition operates and to which it responds in a field as obviously mediated by politics and capital as social media (Iqtidar 2011; Ahmad 2009; Islam 2015; Yavuz 2004). This also pertains to notions of the secular state of modernity itself (Hirschkind 2006; Asad 2003). For Husaini, the pious self at stake is fundamentally practical, as it addresses questions of time, decency, virtues, and duties (all organized around a morality that involves a relation to scripture as much as a sensitivity towards the kind of new digital phenomena – e.g., highly emotional and transgressive forms of “extreme speech” [Pohjonen and Udupa 2017]) that may come up for the health of the soul (its ultimate relation to God) of those who engage in *dawat*. It is a self that also fits within the framework of minority politics related to moderation qua legalist and constitutional discourse (the latter discourse having been identified by Hilal Ahmed [2019] as dominant within most politically active Muslim groups in India).

## Conclusion

[T]he fundamental question whether a global media ethics is indeed possible in a world marked by difference is a theoretical one. (Christians et al. 2008, p. 137)

If posed like this, it will and, indeed, *must* remain a theoretical question. What are differences, after all? Culture, society, tradition, nation, history, region, locality, gender, and religion – all of these terms can be seen as a source of difference. Sometimes difference acquires the proper abstraction of exchange value: Pick your favorite term of comparison; it is *your* choice – at least *in theory*. This abstraction from tradition which also marked a protestant/capitalist moment in ethics (MacIntyre 2002, 122) is in my own understanding both necessary and impossible. It is necessary to consider the contingency of difference (the theoretical moment of post-foundationalism) while acknowledging the impossibility of establishing this as itself being a foundation. Thus, paradoxically, only by acknowledging the need for abstraction (*abstrahere* etymologically means to detach, pull away, and divert) may we – those invested in meaningful dialogue – engage more deeply with multiple traditions. This is linked to the historical impossibility of solving the artificial division between ethics (theory) and morality (practice and order) in a world rife with contradictions. To sum up: My argument is that this latter difference cannot be resolved through deliberation (conceived as values or principles) meant to *serve* as a foundation for the “global” in media ethics. Ethics, morality, and politics rather begin with trouble – the real kind of trouble that human beings encounter in a globally connected, volatile, and increasingly digitalized environment. That is also why theoretical and empirically driven approaches cannot be properly differentiated and nor can there be any stabilized form of a *global* media ethics.

The social context of the field of global media ethics is, of course, delimited by the position of academic labor within information capitalism. That means that ethics remains conceptualized *within* a domain that may speak about political economy, but is not touched by it in any existential (and conceptual) way. This holds true also for Sadatullah Husaini’s critique of capitalism – or at least he does not develop how the social conditions of people’s life relate to authority in scripture. While the field of global media ethics asks relevant questions, their answers are guided by unnecessary limitations derived from the conceptual framework itself (e.g., by focusing on *ethical principles*). By this, I mean the very attempt to ontologically ground a global media ethics in concepts such as relationality, humanism, concern for life, virtue or care ethics, and overlapping proto-norms. The exclusion of Islamic thinkers outside transatlantic academia from the debate mirrors Husaini’s engagement with transatlantic theory. He neither explores in depth the sources that could trouble the Jamaat’s consensus nor does he take account of the changed academic reflection on digital technology in the last ten years (some of his arguments could have been strengthened by reading Christians’ recent book [2019] on digital media and ethics). His approach contains a structural critique that is similar to those of the field of global media ethics in the sense that it takes the moral domain as primary to structural constrains. His mixture of virtues and duties may speak to the critique of liberalism offered by most global media ethics scholars (however, not to those liberal voices that address Islam within the larger media and journalistic-ethics corpus).

The contradictions that emerge are exclusions in the name of pluralism on the side of global media ethics because it always requires the idea that differences can be accommodated in a framework instead of encountered in the open. On the other hand, in “Cyberistan”, the assumption of “real reality” is linked to an instrumentalist approach that is part and parcel of modern scientism. The latter has, however, perpetuated the very subject-object dilemmas in European modernity – including liberal individualism – that Husaini tries to overcome with an Islamic cure.

## Footnotes

1 The term is not equivalent with “the West”. It does not try to comprise or *other* all that goes on in formerly colonizing countries of the global north. Instead, it refers primarily to English language scholarship within neoliberal academia. Thus, it is perfectly possible to say that there are authors based in India who participate in *global* transatlantic academia. To a lesser degree, this area also includes scholarship in other European languages and – though much less so – in South Asian “vernacular” languages. This paper is itself “transatlantic” in the given sense.

2 Perhaps I offer a negative approach but whether this is an “approach” which can be “offered” is debatable (see my discussion in the section “limiting ethics”).

3 Even though the latter point is rather problematic, in that it tries to establish a distance towards a religious *other*, I consider some *Aristotelian* questions concerning everyday practice of major importance for many Islamic traditions and their approaches towards virtuous behavior. Saba Mahmood (2012) traces this anthropological tradition via Talal Asad back to Michel Foucault. It stresses the study of a “set of embodied capacities and dispositions that one acquires through labor in order to become the willing subject of a tradition” (Mahmood 2012, 225). Interestingly, questions of *Aristotelian* virtuous disposition are also key to some recent thinking on “digital ethics” (Grimm et al. 2019; Ess 2014).

4 In fact, the range of philosophical traditions Christians draws upon in his recent book *Media Ethics and Global Justice in the Digital Age* (2019) makes clear that his approach seeks to join and connect rather than to challenge or critique. His expansive mode links thinkers via a *decontestation* of their concepts (by limiting the internal coherence of concepts within the work of these widely differing thinkers). For example, he discusses *aletheia* (the Greek word for the state of being disclosed) in relation to Heidegger, St. Augustine, ‘Hinduism’, Gandhi, Derrida, and so on (ibid 159–168).

5 Nick Couldry, for example, is rather renowned for his important contributions to cultural studies and our understanding of practices of mediation and processes of mediatization (Couldry and Hepp 2017).

6 For example, Couldry (2016, 107) criticizes Christians’ Christian humanism.

7 My understanding of “the political” (as opposed to institutionalized politics) follows the Essex school’s understanding of an ontological category: the political is the contingent grounding of social order through an antagonism (Marchart 2018).

8 This is actually a rather old insight that has been elaborated upon by some of the founders of modern sociology such as Karl Marx (West 2008, 63–101) and Emile Durkheim (Giddens 1972, 31) reaching back in more notional forms to Rousseau (MacIntyre 2002, 180) and Montesquieu (Fromm 1977, 22). It depends on how ethics is fleshed out within social relations while acknowledging the *Machiavellian* moment (MacIntyre 2002, 125), in that ethics and politics are connected and dependent on the contingency of political orders (see section “Limiting Ethics”).

9 It is helpful to read Islamic Studies authors (Brown 2015; Rohe 2011) in order to get a glimpse of the complex traditional protocols and the variety of legal schools that connect scholars (*ulema*) to the normative sources of Islam.

10 This again brings us back to the difficulty of translating *din* as *religion*. Islam does not have an organized church and it is not possible to graft many of its traditions (legal and mystical) onto a protestant understanding of religion that prevails in much of scholarship: “When we conceive of Islam as *religion* we are ineluctably entering into a conceptual order(-ing) – into a taxonomy of ideas, actions, and objects – that assumes the universality of the Modern Western religious-secular binary. Simply, the adoption of the pseudo-universal category religion implicates us in the pseudo-universal category secular.” (Ahmed 2015, 197).

11 Among modernists and reformists, there still exists a wide range of reading strategies that often take account of earlier scholarship on the normative sources of Islam. “Quran only” approaches (Brown 2015) mark only one rather extreme position within these groups.

12 *Dawa* (or *dawat* in the Indian and *dakwah* in the Southeast Asian context) refers to the proselytizing, preaching, and propagation of Islam (I will give a more detailed account of these practices below).

13 I do not claim that Rao and Wasserman are representative of the diverse body of works that go under the name of post-colonial studies; they are only prominent in making the link between some of these works and global media ethics.

14 When the human subject is feeling lost, it is dislocated by radical contingency (Glynos and Howarth, 2007). In response, there are different ways of coping, negotiating, and challenging social reality. This implies that politics, ethics, ideology, and social structure cannot be studied apart from each other. They rather need to be seen in relation to how subjects respond to the changes and problems that enter their lives.

15 The critique advanced here is not new. Already, Aijaz Ahmad (1992, 15) pointed out that, among some post-colonial literature, *brahmanical* terms (such as *ahimsa*) are combined with European theories (especially those of Foucault) to idealize an Indian self without proper socio-political contextualization of the position from which these theories emerged, nor establishing a rigorous conceptual building through which analysis may become accountable.

16 See the critique of Irfan Ahmad (2017) regarding a similar maneuver in Amartya Sen’s “The Argumentative Indian” (2002). He argues that the latter’s work has an implicit nationalist agenda in that it ignores critical protocols of debate developed among Indian Muslims. Taking Sanskrit-based terminology as a metonymy for “Indian” conceptual traditions is thereby always a problem as long as the boundaries of scale, reach, and efficacy of these concepts are not developed.

17 I take it for granted that there are many other post-colonial approaches in what is a diverse field. Rao and Wasserman, however, speak to global media ethics and are therefore discussed here.

18 Vivek Chibber’s (2013) critique of Dipesh Chakrabarty (who is quoted by Rao and Wasserman) is illuminating regarding the question of what concepts attempting to provincialize Europe actually refer to. Is it the grand narrative of European modernity or is it the possibility to critically explain political and economic processes in terms that have been historically formed in Europe but are applicable to those processes wherever they may occur (e.g., commodification)? Chibber argues that it is incoherent to provincialize Europe regarding those second phenomena that are actually transregional. One may paradoxically end up homogenizing and reinstating the special features of “Europe” and “India” while at the same time denouncing “grand narratives” (ibid 126–129).

19 Already, Marx stressed the need to see theory as a *response* to questions that history raises (West 2008, 63–101). His understanding of what is just and right always took account of how the antinomies of morality are *practiced* within conditions of historical contingency. However, the reason why I do not squarely place my argument in the Marxian position is that he again founds history on the mode of production. This is, indeed, a critical position that enables one to investigate the limits of liberal polities as they are confronted with surveillance capitalism (Zuboff 2019) and its economical undermining of institutionalized core values. It nevertheless does not enable one to account for a radical engagement with difference and a radical openness towards contingency (Laclau and Mouffe 1985).

20 The latter rejection would rather mark the kind of work often parodied under terms such as “radical constructivism” or “post-modernism”, where claims about knowledge supposedly delve into an all-out relativism. These polemics are, however, of limited use since they tend to overlook the variety of constructivist claims about the contingency of knowledge coming from a wide range of philosophical pedigrees (post-Kantian, psychoanalysis, Marxist, life-philosophical, and existentialist) – all having different methods of arguing around the lack of solid foundation without necessarily denying that some things are better founded than others.

21 Suspicions and doubts about the Jamaat’s nationalism abound in India, and the ban of the former students’ wing of SIMI as a terrorist organization in 2001 (extended in 2019) and the recent exile of popular TV-preacher Zakir Naik have surely contributed to general caution regarding organizations that engage in *da’wa.*

22 In this section I extend on material that I have been using for a yet unpublished article (Udupa, *author* and Siddique, forthcoming).

23 I do not have the exact years, but the references given in the first chapter suggest the early 2000s. The second chapter must have been written either in 2018 or shortly before.

24
https://www.youtube.com/watch?v=SjIR6wbOY9w&t=1s (access 09.03.2020).
